# Toward improved stroke care in Nepal: insights from a qualitative study on barriers and success factors

**DOI:** 10.3389/fneur.2025.1562948

**Published:** 2025-08-07

**Authors:** Christine Tunkl, Raju Paudel, Lekhjung Thapa, Shirsho Shreyan, Alexandra Krauss, Ashim Subedi, Balgopal Karmacharya, Pankaj Jalan, Nima Haji Begli, Patrick Tunkl, Sunanjay Bajaj, Andrea Kosinski, Christoph Kosinski, Pradesh Ghimire, Bhupendra Shah, Avinash Chandra, Mahesh Raj Ghimire, Bikram Prasad Gajurel, Jessica Golenia, Jan van der Merwe, Christina Stang, Rupal Sedani, Christoph Gumbinger

**Affiliations:** ^1^Department of Neurology, University Hospital Heidelberg, Heidelberg, Germany; ^2^Grande International Hospital, Kathmandu, Nepal; ^3^National Neuro Center, Kathmandu, Nepal; ^4^Rajshahi Medical College, Rajshahi, Bangladesh; ^5^Manipal Teaching Hospital, Pokhara, Nepal; ^6^Norvic International Hospital, Kathmandu, Nepal; ^7^Tunkl Consulting, Heidelberg, Germany; ^8^Department of Neurology, McGovern Medical School, University of Texas Health Science Center at Houston, Houston, TX, United States; ^9^Praxis Meertens, Geilenkirchen, Germany; ^10^Rhein-Maas Klinikum, Teaching Hospital University RWTH, Aachen, Germany; ^11^Bharatpur Hospital, Bharatpur, Nepal; ^12^B.P. Koirala Institute of Health Sciences, Dharan, Nepal; ^13^Annapurna Neuro Center, Kathmandu, Nepal; ^14^Devdaha Medical College, Devdaha, Nepal; ^15^Tribhuvan University Teaching Hospital, Kathmandu, Nepal; ^16^Angels Initiative, Ingelheim, Germany; ^17^Health Management Institute z.u., NGO, Brno, Czechia

**Keywords:** stroke, Nepal, implementation research, qualitative research, public awareness, quality monitoring, stroke care advocacy

## Abstract

**Background:**

The Nepal Stroke Project (NSP) aims to improve stroke care in a resource-limited setting. This qualitative study explores healthcare professionals’ experiences and perceptions of barriers and success factors in implementing stroke care in Nepal.

**Methods:**

Semi-structured interviews were conducted with eight healthcare professionals (six physicians, two nurses) involved in the NSP. Interviews were analyzed using qualitative content analysis, following a constructivist approach.

**Results:**

Success factors encompassed the dedication of healthcare professionals, involvement of hospital boards, effective training initiatives, and the formation of stroke teams. Positive developments noted were increased thrombolysis availability, improved stroke awareness, and growing interest in stroke care among medical professionals. Key barriers identified included lack of government ownership in stroke care advocacy, financial constraints for patients, inadequate public awareness, and challenges in implementing quality monitoring.

**Conclusion:**

While the NSP has initiated positive changes in Nepal’s stroke care landscape, significant barriers persist. The study highlights the importance of addressing systemic issues such as government involvement and financial accessibility of treatments. Success factors, particularly the motivation of healthcare professionals and local ownership of the project, provide a foundation for future improvements. These findings can inform strategies for enhancing stroke care delivery in other resource-limited settings and guide ongoing initiatives within the NSP.

## 1 Introduction

Stroke presents a significant global health burden, with a disproportionate impact on low- and middle-income countries (LMICs). Despite bearing 86% of global stroke deaths and 89% of disability-adjusted life years lost, LMICs face substantial challenges in implementing evidence-based stroke care practices (1). While theoretical frameworks exist (2–4), their practical implementation remains challenging. The disparities between LMICs and HICs in stroke care are well studied, revealing a paradox wherein LMICs bear the major burden of strokes yet suffer from limited access to and high costs of treatment (4–6). Nepal exemplifies this scenario with a high stroke burden amidst a resource-restrained healthcare infrastructure (7). Since 2021, the Nepal Stroke Project (NSP) stands out as an initiative to tackle these unmet needs (8). Health care professionals (HCPs) play a critical role in the implementation of different strategies in everyday health services and their experiences are essential to inform the development of effective strategies for improving stroke care delivery. Our research with a qualitative approach aims to delve into the experiences, perceptions, and attitudes of healthcare professionals involved in the process of stroke care transformation in Nepal. By elucidating the challenges encountered (“barriers”) in clinical work and the factors leading to successful implementation, we aimed to identify specific areas for continuous improvement. Our findings will not only inform the ongoing initiatives within the NSP but also contribute to broader organizational-level improvements in stroke care delivery in resource-limited health care settings.

## 2 Methods

### 2.1 Design

This qualitative investigation utilized semi-structured interviews to understand the perceptions of health care professionals (HCP) toward the Nepal Stroke Project (NSP). The study embraced a constructivist perspective, purposefully integrating researchers’ personal experiences and expertise to heighten theoretical sensitivity and foster theory development. This approach was chosen based on its appropriateness for delving into perceptions and comprehending the factors having shaped the project (9).

The researcher CT, who served as both a co-founder of NSP and project coordinator from its inception, maintained a stance of reflexivity throughout the study, consistently reflecting on their assumptions and biases (10). This reflexivity ensured a conscious awareness of how her background and involvement might influence the interpretation of data and the development of insights.

### 2.2 Setting

The NSP, initiated in 2021 by the Nepal Stroke Association in collaboration with the University Hospital Heidelberg, aims to improve access to quality stroke care (8). The project aims to establish stroke-ready hospitals and nine secondary or tertiary care centers in different provinces and in the capital Kathmandu were actively participating in the projects’ activities. During the first 2 years, more than 1,000 healthcare professionals attended stroke-related workshops (8), and quality monitoring training through which Registry of Stroke Care Quality (RES-Q) was introduced (11). Awareness campaigns reached over 3 million individuals through social media and live events (12).

### 2.3 Study participants

Participants were identified using purposive sampling to ensure the collection of data from individuals likely to offer valuable insights into the strategies employed. The sample comprised health professionals actively engaged in the NSP. Maximum variation sampling was employed to select participants representing a diverse range of perspectives, experiences, and roles within the project. This approach encompassed individuals involved from the project’s inception as well as those who joined later stages. The sample included health professionals from both regional hospitals and the capital, ensuring representation across varied healthcare settings. Participants ranged from junior to senior positions and encompassed a mix of physicians and nurses, enriching the study with a breadth of expertise and viewpoints.

### 2.4 Participant recruitment and data collection

Potential participants were invited to participate in the study by a research assistant (AK) utilizing a stepwise recruitment process. To ensure confidentiality, interviewees were pseudonymized, aiming to mitigate the influence of social desirability bias. All interviews were conducted remotely via an online meeting platform (Google Meet) and recorded in both audio and video formats for accuracy. Transcriptions of the interviews were completed verbatim, and researchers solely worked with transcripts to prevent depseudonymization. To minimize power imbalances, the interviews were conducted by a research assistant (SS), a last-year medical student from a geographical near-by country, who has not been involved in the projects’ activities before. A semi-structured interview guide, honed through two separate pilot interviews with the research team, provided structure and consistency to the interview process. Recruitment and data collection was performed between June and August 2023.

### 2.5 Data analysis

To ensure analytic rigor, thematic saturation was evaluated continuously and considered achieved when no new themes emerged, and interview responses began to repeat. This decision was confirmed by two researchers.

Data analysis followed the approach of qualitative content analysis as outlined by Kuckartz (13). Coding rules were initially developed deductively, based on existing theoretical frameworks and the interview guide.

The analysis commenced with one author (CT) conducting an initial review of the transcripts, noting preliminary observations and thoughts, followed by a line-by-line analysis of each transcript to identify initial codes, which were then clustered into related categories. Codes identified through this deductive process were refined and grouped inductively into overarching themes related to barriers and success factors.

**Figure 1:**
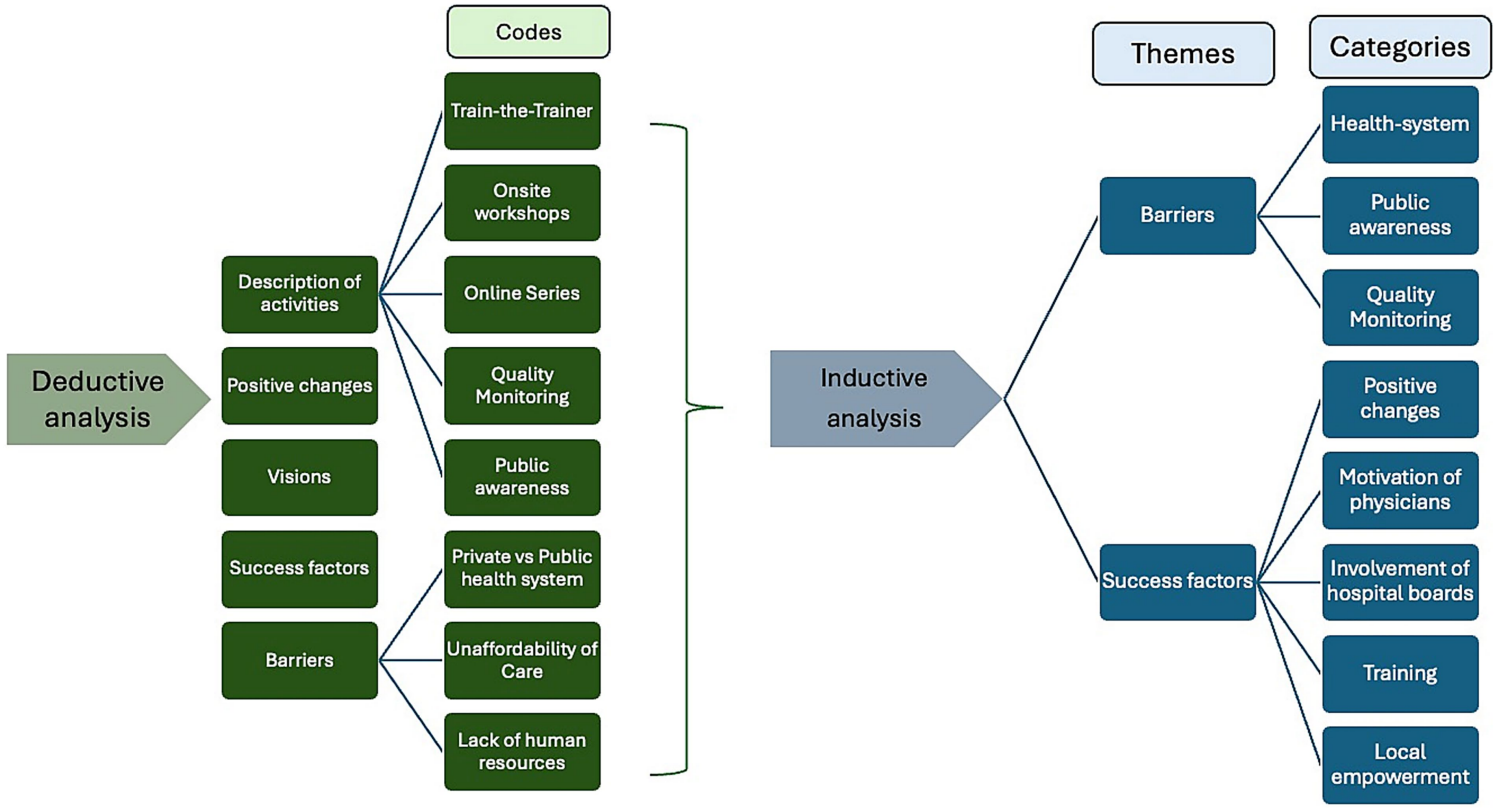
Codebook structure depicting key themes in stroke care delivery in Nepal.

For coding validation, interview transcripts were independently coded by a researcher proficient in qualitative interview research (CS). Each researcher developed a coding framework; these were compared, found to be highly similar, and then merged into a final codebook through discussion and consensus. Categories were defined with anchor examples from the transcripts to guide consistent application. This process enhanced intercoder reliability and transparency (see Figure 1). The analysis was presented and discussed at a Qualitative Journal Club to enhance validation of findings. To support the analysis of the transcription material, the MAXQDA software (VERBI, 2018) was used. Adherence to the Standards for Reporting Qualitative Studies (SRQR) ensured methodological rigor throughout the analysis process (14).

### 2.6 Ethical considerations

Ethical approval was obtained from the ethics commission of the Medical Faculty of Heidelberg University (S-238/2023). Prior to participation, interviewees provided informed consent, ensuring compliance with ethical standards. During the interview, they had the opportunity to interrupt the interview at any time. It was also always possible to withdraw consent retrospectively.

## 3 Results

### 3.1 Study participants

We conducted interviews with eight participants, who comprised six physicians and two nurses, for details see Table 1. The participants were affiliated with diverse healthcare institutions across various cities in Nepal, reflecting the project’s reach and engagement with stakeholders across multiple regions of Nepal (see Table 1). The average time of the interviews was 52 min (ranging between 17 and 95 min).

**Table 1:** Information on study participants.

	Pseudonym	Profession	Hospital level of specialization	Hospital funding	Location
1	Pallav	Emergency physician	Tertiary care facility	Private owned	Outside Kathmandu
2	Saatvik	Consultant Internal medicine	Tertiary care facility	Private owned	Outside Kathmandu
3	Chandrashekhar	Consultant Neurologist	Tertiary Care Facility	Private	Kathmandu
4	Uday	Neurologist	Tertiary Care / University Hospital	Government-owned	Kathmandu
5	Eshaan	Emergency physician	Tertiary care facility	Government-owned	Kathmandu
6	Jagadeep	Neurologist	Tertiary care facility	Private	Kathmandu
7	Garima	Nurse	Tertiary care facility/University Hospital	Private	Kathmandu
8	Aditi	Nurse	Tertiary care facility	Government owned	Outside Kathmandu

### 3.2 Overarching categories and themes

#### 3.2.1 Theme 1: barriers in implementing stroke care

Barriers were found in the areas of stroke care advocacy (1) (at a health-system level), public awareness (2) and quality monitoring (3) (see Table 2).

**Table 2:** Barriers identified within the Nepal Stroke Project with quotes.

Barrier	Quote	Interviewee
Stroke care advocacy		
Unavailability and unaffordability of thrombolytic medication	Still there are patients who cannot afford the medication. We try to convince them that life is more than the cost of the medication.	Pallav, 73
	Our people cannot afford disability. They would rather die.	Pallav, 110
	Our people do not have insurance. Our government does not support other people. So, you’d rather die than getting the treatment that would be the best option for them.	Pallav, 110
	One of the challenges for implementing all these treatment strategies again the cost factor because most of our patients they have to pay out of the pocket.	Chandrashekar, 163
	The medicines are very expensive, so they cannot afford [.] and at other times the people are not ready, the team is not ready, so we run out of the time. [.] The major factor is definitely cost.	Eshaan, 140
	When we started the availability of drug was a question. Sometimes the drug was available sometimes not, [.] when the patient was ready, there was no, when drug was available, patient was not ready [.]. Only because the patient had to pay and the hospital could not keep the medication for a longer period of time because it’s expensive medication.	Pallav, 67
	The biggest problem is the affordability [.] in government hospital and [.] in private also [.]. Because whenever the patient will come and if we have to thrombolyse the charges from the MRI to the lab and the treatment part goes very high. So affordability is the biggest challenge that we have in both setting.	Jagadeep, 156
Lack of human resources	I make a team, then three to 6 months, they can get changed and we have to say, again, Make a team.	Saatvik, 63
	The problem is rapid turnover of [health care personnel] from our country. They take the few months training session and they will [go] abroad […].	Saatvik, 107
	It’s very difficult to keep the team intact for a long time. We always have people who are just moving on to a different country or leaving the jobs—that becomes a big challenge for us.	Chandrashekar, 75
	One of the biggest challenges in stroke care is having a good stroke team. [.] And as a developing country, I mean, one of the biggest challenges is again, those turnarounds, the turnovers. [.] That makes it very challenging to implement all these strategies because it takes time for new people to learn the things.	Chandrashekar, 134
	Many doctors on Friday or on Saturday will go to the remote places we call as a remote clinics.	Jagadeep, 61
	Our emergency used to be seen by the medical officers and the interns mostly. And I was the only consultant when I started.	Pallav, 8
Discrepancy between private and public sector	Public and private partnerships would be the main core of the stroke management and the involvement of the government still will give the opportunity to infiltrate into the government system and strengthen this project in the government hospital also.	Eshaan, 32
	The resources in private hospital is better than in government hospitals. The way of management is more swift in private than the government hospital.	Jagadeep, 56
	In the Government Hospital […] they have a kind of supremacy attitudes. So they do not want to change or they do not want to accept anything for positive.	Jagadeep, 142
Government must take responsibility	We need to connect with the government system and the government people so that they also take the ownership. NSP alone cannot take charge of the whole Nepal.	Eshan, 188
	The NSP has not been able to get at the people who are in the administration level or the policy making level though we should think of that also.	Eshan, 167
	We missed the people who are the policy level and what is necessary, is a seminar which concludes with the mandate, and the mandates are handed over to the government. So that type of seminar is necessary in the stroke.	Eshaan, 182
Public awareness		
Believing in healers	We have one Ayurvedic hospital nearby and they are […] treating more patient than us.	Saatvik, 136
	They first seek for the spiritual faith, I think 90% of patients better go to lama or healer and we only see 10% of stroke in our area in medical hospital.	Saatvik, 134
	Many patients in Nepal do not come to hospital because they feel they get better tomorrow. One of the studies we done, the average time to presentation to hospital was 29 h and last year we did one study, that was still 24 h.	Uday, 147
More needed from NSP	And on the other hand I think there should be more public awareness, […] and Nepal Stroke Project needs to tie up the national system of public awareness. There are lots of platform in the national system.	Eshaan, 180
Misperceptions	. it might cause bleeding in the brain and [.] my patient dies because of the medication [.]. Such an expensive medication that could cause that rather not to use the medication. This sort of things that is being implemented by our health professional itself.	Pallav, 267
Quality monitoring		
Uncertainty about data security aspects	Quality monitoring is not possible unless and until policy and administrators pitch in. Quality monitoring cannot be done by the clinician.	Eshaan, 177
	We cannot put in the data in any quality monitoring database without the permission. So when it comes to the permission, it should come from high level to us.	Eshaan, 159
	I came to know that the person who is in charge of all those RES-Q can have access to every data from every country. I am not very comfortable with the data in that manner.	Jagadeep, 160
Lack of time and not mandatory	People are so busy, they will not enter the data so it will not be possible unless the policy and the administrative people coming to the scenario.	Eshaan, 179
	The thing is that it’s not mandatory and when there is no mandatory and no reward or no punishment for that, it does not generate interest in people […].	Jagadeep, 173
Complexity of the tool and workflow	The biggest hindrance I think is going back and sitting on the computer and just entering the data. […] If there could be a RES-Q app on the phone […] when we are doing the rounds in front of the patient.	Chandrashekhar, 179
	Still feel very complicated using RES-Q.	Jagadeep, 159
	We do not have Internet service during the daytime, I just getting internet after seven pm right now.	Saatvik, 406
	Even in my hospital we do not keep follow-up data. We have a record system. We give that to the patient and the patient takes that record with them. We do not keep that in the hospital. So, it’s very difficult to keep follow-up data of the patient […].	Uday, 116
	We tried to involve some of our students to enter data [.] and we found that it’s very difficult to […] sustain that because students keep on changing and […] RES-Q registry requires training.	Uday, 116
Possible solutions	If we could develop an app based RES-Q entry for entry app. I think that would be very effective tool. […] A RES-Q app on the phone, where we could just fill it up and right, when we are doing the rounds in front of the patient.	Chandrashekhar, 182
	Maybe we can use some simple apps which can be mobile-based apps which can be used to enter data also. [.] Just the deficits, the type of tests used and the type of discharge medications, the time of follow-up. I think that would be a better idea to start with.	Uday, 129
	Another option is to keep a research officer. [.] The problem is with funding. But because that would help improve stroke care in a hospital, that’s another way of including stroke care in a hospital. [.] Because a research officer will not only do RES-Q in the registry data entry. Research officer can help with other research activities.	Uday, 126

##### 3.2.1.1 System-level barriers

Interviews described a lack of government ownership in the field of stroke care as a significant challenge. Participants consistently emphasized the limitation of the NSP to effectively influence policy making, highlighting the necessity for broader engagement by the highest level of health authorities.

With the lack of health authorities taking responsibility comes that financial constraints were identified as a significant barrier to accessing stroke care. Patients are often unable to afford necessary treatments in stroke care, which has to be paid out-of-pocket:

“Our people do not have insurance. Our government does not support other people. So, you’d rather die than getting the treatment would be the best option for them” (Pallav).

The unavailability of essential and time-critical thrombolytic medication aggravates this, as one physician describes:

“Sometimes the drug was available sometimes not, [.] when the patient was ready, there was no, when drug was available, patient was not ready” (Pallav).

Disparities between private and governmental hospitals were highlighted, as well as in access to stroke care resources between the capital Kathmandu and the provinces.

High staff turnover rates and difficulty in forming cohesive stroke care teams were noted as significant obstacles with a lack of experienced consultants and mostly emergency departments being run by medical officers.

##### 3.2.1.2 Low level of stroke awareness

Low public awareness poses a significant barrier to effective stroke care, with misperceptions, and notable trust in traditional healers and spiritual health, especially described by HCPs from rural areas:

“We have one Ayurvedic hospital nearby and they are [.] treating more patient than us” Saatvik.

Erroneous beliefs are prevalent also among physicians, particularly regarding thrombolytic medication, as still many physicians fear administering this treatment due to concerns about increased bleeding risk.

##### 3.2.1.3 Quality monitoring

All participants acknowledged challenges encountered with hospital-based quality monitoring during the initial implementation efforts.

*Hospitals’ workflow*: Major challenges were noted in the hospital’s workflow, as records are taken home by patients:

“In my hospital we do not keep follow-up data. We have a record system. We give that to the patient and the patient takes that record with them. We do not keep that in the hospital” Uday.

*Non-Mandatory Nature and Time Constraints*: Participants highlighted the non-mandatory nature of RES-Q in Nepal and the time constraints faced by clinicians as significant barriers. One physician emphasized the need for not only training clinicians in quality monitoring, but also called for policy and administrative support. Others echoed this sentiment, stating that “it’s not mandatory and when there is no mandatory and no reward or no punishment for that, it does not generate interest in people.” (Jagadeep). As one participant stated, “People are so busy, they will not enter the data so it will not be possible unless the policy and the administrative people coming to the scenario” (Eshaan).

*Data security aspects*: Physicians from governmental hospitals cited a perceived lack of permission from high governmental authorities as a justification for not using the RES-Q database. While terms and conditions of RES-Q are clearly defined, physicians expressed concerns about uncertainty regarding who could access the registered data.

*RES-Q online tool*: Limited internet access in some hospitals was mentioned as a further barrier to regular use of the online data entry. Participants expressed difficulty in navigating the online tool of RES-Q.

“The biggest hindrance I think is going back and sitting on the computer and just entering the data” Chandrashekhar.

Participants suggested potential solutions, including the development of a mobile app for data entry that can be used instantly. Another proposed solution was to appoint dedicated research officers in each hospital responsible for RES-Q data entry, as it was mentioned that training students had proven unsuccessful.

#### 3.2.2 Theme 2: positive developments and success factors

We identified positive advancements in stroke care, public awareness and stroke care advocacy. Success factors enabling these changes included the dedication of health care professionals and involvement of hospital boards, training initiatives, and the formation of stroke teams. For details see Table 3.

**Table 3:** Positive changes and success factors with quotes.

Factors	Quotes	Interviewee
Positve changes	I think the project has really changed the stroke scenario in Nepal.	Chandrashekar, 185
	Most people, now they know that there is a drug which they can take.	Uday, 151
	The biggest positive thing that we have now seen, that the thrombolysis itself after NSP has sky-rocketed in Nepal. We have many centers outside KTM who are thrombolysing	Jagedeep, 207
	Most of our nurses, did not know about the stroke, and they did not know that there is management for a stroke […]. And with those workshops. That has changed.	Pallav, 43
	After getting involved with all those people who are treating the stroke day in and out [.] I started to learn stroke and I started teaching how to diagnose. [.] After I started teaching others, we had a good team of the people who were well oriented with the stroke and we started making a protocols and all those things and we started teaching other people also. So in that way, we develop a good network inside the hospital.	Eshan, 143
	Few years back, also, we did not have treatment protocol or we did not have a stroke team but now stroke team is upcoming and the treatment protocol.	Eshaan, 26
	The stroke cases before is to be admitted by the neurosurgery. Now the internal medicine people are interested in history. They are learning more on this to. The residents are getting eager to treat this too.	Pallav, 179
	The government will also help us having some kind of subsidy for a stroke treatment, which is not set yet but at least, the government has shown some positive response. So, these are things that I’m quite satisfied for now.	Jagadeep, 30
Motivation of doctors	I feel that I’m the integral part of this whole project. And I especially like to go out and involve in teaching programs, in the conferences, so that activities make me feel that I have some ownership in this project.	Chandrashekar, 28
	I feel pride, that I got involved in all these activities and I’m getting a chance to advocate at national levels… I’m just a family doctor, but they call me for a lecture there and I feel happy that I was called for that.	Pallav, 91
	I always feel a sense of ownership in everything that I deliver. And in all activities that I have participated with Nepal Stroke Project, [.] I always felt that I have owned and I’m fully committed […].	Uday, 29
	People should be motivated and they should come by their own by heart […], so we need to think about the intrinsic motivation, rather than extrinsic motivation.	Eshaan, 119
	The venue is important […] [because] if they are told that it’s being in a hotel that is quite famous, I think there would be more tendency to go.	Pallav, 119
Involvement of hospital boards	The new administration wants to just build up the hospital in a new level. [Stroke] was one of the opportunities for them, so we try to hit the iron when it was hot.	Pallav, 71
	We get support all type of support from our hospital because now, everybody has learned that stroke is such an emergency.	
	We advocated to our administration and we just encourage them to keep at least one or two valves of medication […] so it’s available at this time regularly. And they are keeping it.	Pallav, 69
	Once Nepal Stroke Project was taking stroke more seriously in the hospital and [the hospital’s health care providers] perceived this as [.] someone is monitoring the quality of our stroke care. [.] The emergency people started taking strokes very seriously and this notion that we need the door to the needle time should be shortened as much as possible, that became remarkably short.	Chandrashekhar, 156
Establishing a stroke team	We have a very good stroke team of residents, internal medicine residents and neurology residents, we find it very useful.	Uday, 35
	We have a concept of making a team and let us work together in a team.	Saatvik, 68
	We do have a stroke team in our hospital which include radiology, emergency physician, neurologist.	Garima, 11
Ownership of the project	Because of our commitment it will be sustainable and we will be committed to make it sustainable in the future. So that even when our team of our team members from Germany would leave [.] we would make sure that all these activities will be carried out in the future.	Uday, 20
	Once we get used to all these activities, it’s probably very difficult for things to not get sustained. [.] Because with time and more people we’ll get more awareness and with awareness we’ll be able to get more funding and with more funding we should be able to sustain the activities that are being carried out at present.	Uday, 22
	I think the activities needs to keep going [.] and I think, the more we will take next couple of years to really make this active with the project sustainable. [.] We need to have more, meetings and put in our effort to keep this project sustainable but at this point of time I do not think so. [.] We need to continue activities.	Chandrashekhar, 25
	I do not find that there was any kind of imbalance between foreigners or locals.	

##### 3.2.2.1 Positive changes

The participants highlighted positive developments in stroke care, noting the emergence of a stroke movement in Nepal. Intravenous thrombolysis (IVT) has expanded to many centers outside the capital, with one participant stating it has “sky-rocketed in Nepal” (Jagadeep). Awareness of stroke medication also increased, as “most people now know that there is a drug which they can take” (Uday). Clinicians observed a rise in knowledge of HCPs, evidenced by the establishment of stroke teams and the implementation of protocols and flowcharts for stroke care. Additionally, there has been a surge of interest in stroke care from other disciplines, accompanied by a growing trust in stroke care services.

Although all participants urged for more governmental support, one participant expressed satisfaction with the government’s responsiveness, citing potential subsidies for stroke treatment as an example.

##### 3.2.2.2 Motivation of physicians

Participants expressed pride in their involvement with the NSP, particularly those in the founding group, citing a sense of recognition and ownership in promoting stroke care.

“I feel pride, that I got involved in all these activities and I’m getting a chance to advocate at national levels… I’m just a family doctor, but they call me for a lecture there and I feel happy that I was called for that” (Pallav).

While acknowledging the importance of personal motivation and the identification of local “stroke heroes,” participants recognized that sustainability hinges on broader involvement from health professionals. They stressed the imperative of elevating the program to the health administration and policy level to ensure long-term sustainability.

Moreover, participants identified various factors beyond intrinsic motivation such as pleasant venues, encouragement from senior colleagues, opportunities to attend conferences abroad, and accreditation.

##### 3.2.2.3 Involvement of hospital boards

Participants reported success in engaging hospital leadership by framing stroke care as an urgent and strategic opportunity. This led to support for stroke units and essential medications.

“The new administration wants to just build up the hospital in a new level. So stroke, it was one of the opportunities for them, so we tried to hit the iron when it was hot” Pallav.

Some participants highlighted the hospital’s response to external pressures, perceiving themselves as being monitored, which served as an additional motivator for implementing stroke care measures.

##### 3.2.2.4 Training and stroke team

The HCPs emphasized the significant benefit of the program in enhancing their knowledge base and skills, attributing this gain to the educational materials, on-site workshops, Angels’ Initiative Train-the-Trainer sessions, and regular webinars. Outreach to peripheral provinces was seen as particularly valuable.

Formation of multidisciplinary stroke teams was considered a crucial factor to improving care delivery, particularly in environments characterized by high staff turnover rates.

##### 3.2.2.5 Local ownership of the project

Participants emphasized their commitment to sustaining the project beyond individual involvement, highlighting the importance of continuity and momentum. “Because of our commitment, it will be sustainable, and we will be committed to making it sustainable in the future.” Continued engagement and visibility were seen as key to longevity. While ongoing external support was recognized as necessary, participants viewed the balance between local and international involvement as appropriate and did not express concern over external influence.

## 4 Discussion

This study offers a practitioner-centered perspective on the Nepal Stroke Project (NSP), providing insights into the real-world experiences of healthcare professionals engaged in stroke system transformation in a lower-middle-income setting. Our study unveils areas for enhancement within the NSP, with implications extending to stroke care initiatives in diverse global settings.

Key findings reveal that while personal motivation, training efforts, and hospital engagement facilitated progress, major obstacles persisted—particularly limited government ownership, financial inaccessibility, low public awareness, and difficulties in quality monitoring.

### 4.1 Comparison with literature

Comparable findings regarding barriers and facilitators were observed in the setting of Ghana (15). HCPs in Ghana similarly bemoaned the lack of political support as a major obstacle. This alignment suggests that the success factors and areas for improvement we identified may extend beyond our specific setting in Nepal and could potentially be beneficial in other regions undergoing similar initiatives. Our findings align with the Lancet Neurology Commission’s call for pragmatic, context-adapted approaches to stroke care in low-resource settings (4). Similar to the Commission’s emphasis on health system integration, workforce training, and equitable access, our study underscores the need for structured implementation frameworks, local ownership, and multisectoral engagement to overcome persistent barriers.

In contrast to our earlier publication (8), which provided a structured, process-oriented perspective, this analysis offers insight into how implementation is experienced by healthcare professionals on the ground. Core strategies identified previously—such as local leadership, structured training, and institutional engagement—were reaffirmed, while elements like inter-hospital collaboration were less frequently mentioned, suggesting a divergence in perceived relevance between external observers and local actors. However, our data lack the perspective of non-clinical stakeholders, which hinder triangulation within the health system. Future research should integrate those perspectives.

### 4.2 Unresolved issues and implementation tensions

Persistent implementation challenges reflected deeper structural constraints within the health system. While key interventions were introduced, the lack of a standardized, system-wide framework led to variable execution and limited evaluability. The absence of baseline data and clear follow-up mechanisms was seen not just as a technical gap, but as a missed opportunity to institutionalize learning and accountability.

Efforts to introduce structured monitoring tools like RES-Q underscored this tension: though conceptually embraced, their routine use was constrained by infrastructure, staffing, and unclear mandates. This disconnect highlights a recurring implementation dilemma—external partners may emphasize standardized planning and measurement, but local systems often operate under conditions that require more flexible and adaptive strategies.

Similarly, institutional and policy-level engagement remained fragile. Participants’ experiences suggest that without stronger administrative alignment and formal integration into national health planning, even motivated clinical efforts risk remaining siloed. These tensions illustrate the need to embed implementation within broader systems reform—balancing ambition with feasibility, and external frameworks with local ownership.

### 4.3 Implications

To address the identified challenges, the NSP prioritized the integration of RES-Q as a key quality monitoring tool. Despite its global relevance, implementation in Nepal required adaptation. Multiple training rounds and close collaboration with the RES-Q global team helped overcome initial barriers. Data security concerns were addressed through clear communication and the appointment of a national coordinator. Research managers supported hospital teams for several months, and practical tools—such as paper-based data forms—were introduced to streamline entry. These efforts led to improved data quality and acceptance, culminating in one hospital receiving the WSO Angels Initiative Gold Award.

At the policy level, collaboration with national stakeholders was strengthened, and project funding supported a proof-of-concept for thrombolysis procurement—an effort aimed at improving access to critical treatment and highlighting the feasibility of stroke care integration within the broader health system.

### 4.4 Strength and limitations

Our study provides unprecedented insights into stroke care initiatives within the context of an LMIC. The utilization of an external interviewer and pseudonymization was intended to mitigate social desirability bias. Still, interviewees may have used interviews as a platform to advocate for their own priorities and communicate pride of their achievements. The small, purposive sample (*n* = 8) included predominantly physicians closely involved in the NSP, limiting the diversity of perspectives and increased the risk of participation and confirmation bias. The study focused solely on HCPs and did not include the views of patients, administrators, or policymakers. This restricts the depth of system-level analysis and limits the ability to triangulate findings. Our study was limited to the HCPs involved in the NSP in the tertiary care sector and therefore generalizability to the whole Nepalese health sector is limited. Given the lead author’s close involvement in the project, there is a potential for interpretive bias, despite efforts at reflexivity and independent coding. Observations may have enabled another analytic layer, analyzing the experience of the participants in relation to the observations.

## 5 Conclusion

The Nepal Stroke Project serves as a paradigmatic initiative directed toward implementing stroke care within resource-constrained contexts. Encouraging advancements were found particularly in stroke team integration and increased public awareness of stroke. However, major challenges persisted, notably encompassing the dearth of political backing for stroke care initiatives and establishing robust mechanisms for quality monitoring. These findings highlight the critical role of qualitative research in capturing how implementation strategies are perceived, adapted, and sustained on the ground. Given the shared structural and policy challenges across LMICs, the development of stroke systems requires models that are not only evidence-based but also feasible and responsive to local constraints. Integrating qualitative methods into implementation science is critical to achieving this goal. Further research with various stakeholders and a larger variety of perspectives is necessary to incorporate broader perspectives.

## Data Availability

The raw data supporting the conclusions of this article will be made available by the authors, without undue reservation.
